# Treatment of Incisor Root Resorption Associated With an Ectopically Erupting Maxillary Canine: A Case Report With Eight-Year Follow-Up

**DOI:** 10.7759/cureus.71053

**Published:** 2024-10-08

**Authors:** Fujita Yuko

**Affiliations:** 1 Pediatric Dentistry, Kyushu Dental University, Kitakyushu, JPN

**Keywords:** ectopic eruption, maxillary canine, maxillary incisors, prosthetic treatment, root resorption

## Abstract

Ectopically erupted canines have a relatively high potential to cause maxillary incisor resorption. We present the case of an 11-year-old girl with ectopic eruption of a permanent maxillary canine that caused complete root resorption of the permanent incisors. The procedures used included occlusal guidance and crown restorative treatments of the ectopically erupted maxillary canine and prosthetic treatment for the maxillary central and lateral incisors lost due to root resorption. We treated the anomaly and relieved her mental distress over an eight-year period.

## Introduction

The reported prevalence of ectopic eruption of canines ranges from 0.8% to 9.5% [1-6], which is relatively low. In an Indian study, the maxillary canines are the most commonly ectopic erupted teeth (3.38%), followed by the mandibular canines (1.16%) [7]. However, such eruptions lead to resorption of the adjacent maxillary incisor roots, a serious problem in 48-68% of cases [8-10]. This most frequently affects the lateral incisors, followed by the central incisors [9,11,12]. These teeth may require extraction or expensive, time-consuming orthodontic treatment [13,14], causing immense physical and mental stress. Other potentially deleterious sequelae of ectopically erupting canines can include loss of dental arch length, impaired smile esthetics, and disruption of functional occlusion.

This study presents the eight-year management of ectopic eruption of the maxillary right canine with loss of the maxillary right central and lateral incisors due to severe root resorption in an 11-year-old girl. This case reaffirms the importance of early diagnosis of maxillary incisor root resorption associated with ectopic eruption of a maxillary canine and highlights a strategy for long-term oral development when the diagnosis is delayed.

The patient’s parents consented to the use of patient-related information for the purpose of publication.

## Case presentation

**Figure 1:**
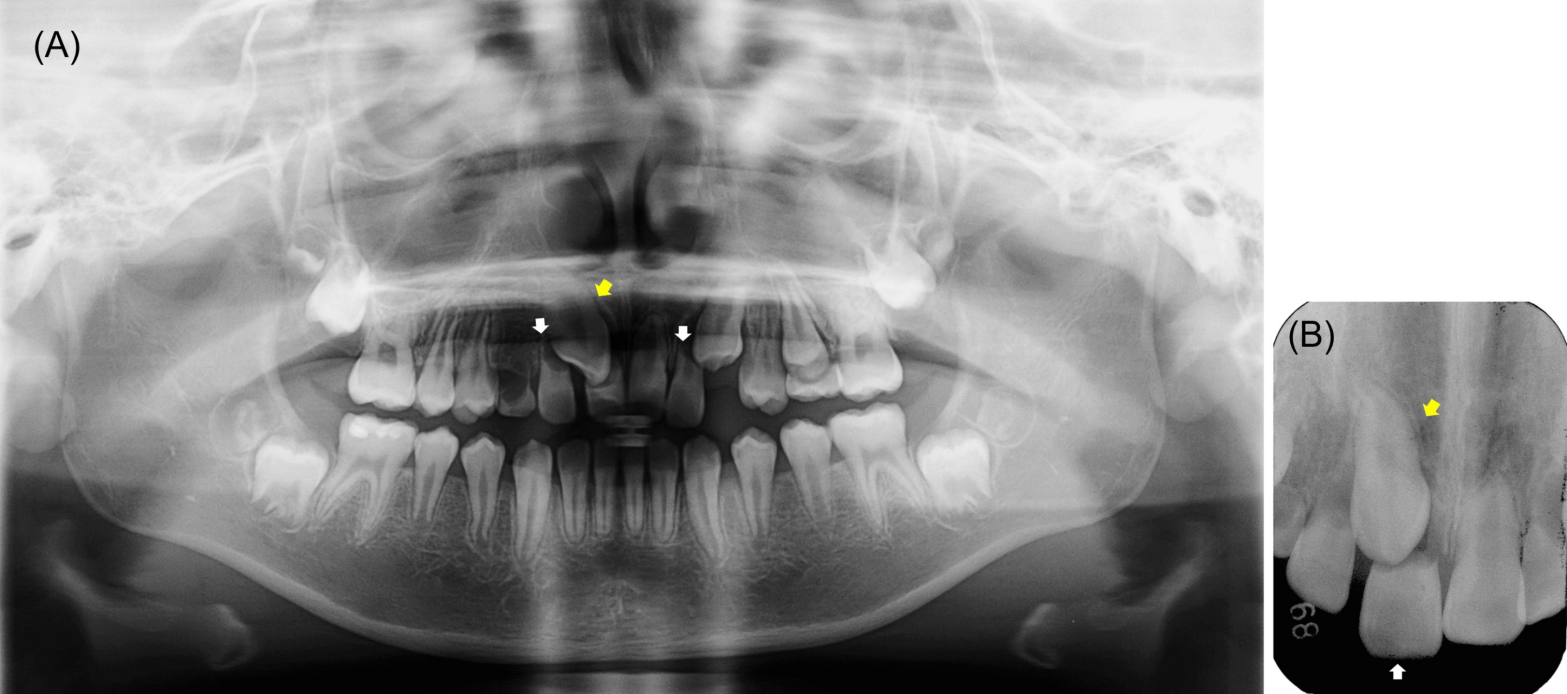
Pretreatment radiographs. (A) Panoramic radiograph. The yellow arrow indicates maxillary right canine impacted directly above the crown of the maxillary right central incisor. The white arrows indicate maxillary bilateral lateral incisors, with more than half of root resorption. (B) Periapical radiograph of the maximally right canine and central incisor. The yellow arrow indicates maxillary right canine. The white arrow indicates the maxillary right central incisor, which showed complete root resorption.

An 11-year, one-month-old girl's initial examination was referred to pediatric dentistry at Kyushu Dental University Hospital from a private dental office in July 2016. The chief complaint was the mobility of the maxillary right central incisor. In the initial interview, she responded that she was very shocked and it was painful for her to talk to other people. A review of her medical and family histories was not contributory. Clinical examination revealed severe movement of the maxillary right central incisor; additionally, the maxillary right canine was palpable above the maxillary central incisor. The degree of mobility in other teeth was within the physiological range. Class Ⅲ malocclusion was evident, with an anterior crossbite. The dentition appeared age-appropriate, except for the retained maxillary right deciduous canine. Radiographic examination revealed that the crown apices of the maxillary left canine were distal to the roots of the lateral incisor, which showed root resorption. The maxillary right canine was impacted directly above the crown of the maxillary right central incisor. Severe root resorption was seen in the maxillary right incisors (Figure 1).

The patient wanted minimally invasive treatment and did not request improvement in her crossbite. It was determined that the incisor could not be saved because the levels of root resorption of the maxillary bilateral lateral incisors and the maxillary right central incisor were severe, affecting more than half of all roots; even traction treatment was performed. We consulted with other specialists in pediatric dentistry, orthodontics, and prosthodontics about her treatment plan and received the same answer as ours: it is impossible to save her anterior teeth with root resorption. They suggested that orthodontic treatment would take several years and could involve risks including root resorption; furthermore, prosthetic treatment would be unavoidable regardless of which treatment was chosen. Therefore, we developed a strategy with the patient and her parents, and the following plan was made. First, the canine would be guided to the position of the incisor. Then the upper right canine would be corrected into the shape of a central incisor. Removable dentures would be used to replace the missing maxillary incisors. Furthermore, we planned to allow her to choose prosthetic treatments, including implants in addition to removable dentures after the permanent dentition is complete. Informed consent was obtained from the patient and her parents before treatment.

**Figure 2:**
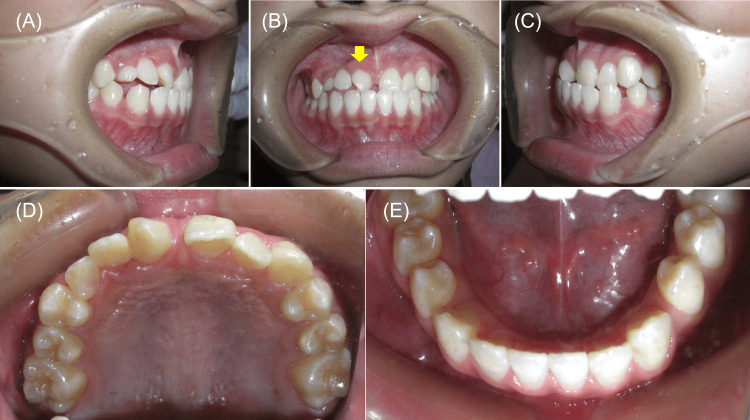
10 months after the initial examination. Intraoral photographs. (A) Right occlusion. (B) Anterior occlusion. The yellow arrow indicates maxillary right canine positioned naturally in the position of the incisors. (C) Left occlusion. (D) Upper arch. (E) Lower arch.

**Figure 3:**
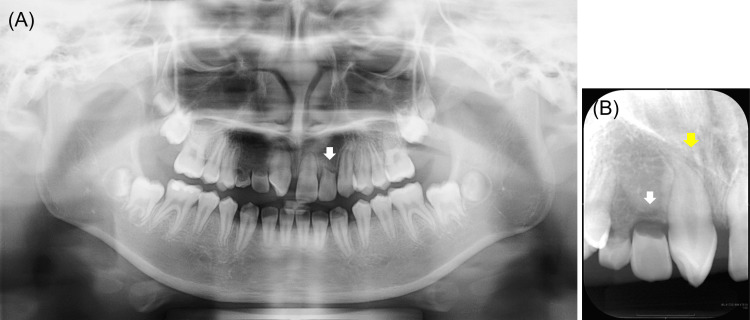
10 months after the initial examination. (A) Panoramic radiograph. The white arrow indicates the maxillary left lateral incisor, which was affected more than half of the root. (B) Periapical radiograph of the maximally right canine in ectopic position in the central incisor region. The yellow arrow indicates maxillary right canine positioned naturally in the position of the central incisor. The white arrow indicates maxillary right lateral incisor with complete root resorption.

Six months after the first consultation, the affected incisor was extracted, and the maxillary right canine was allowed to erupt spontaneously. Four months later, this canine was positioned naturally in the position of the incisors (Figures 2, 3).

**Figure 4:**
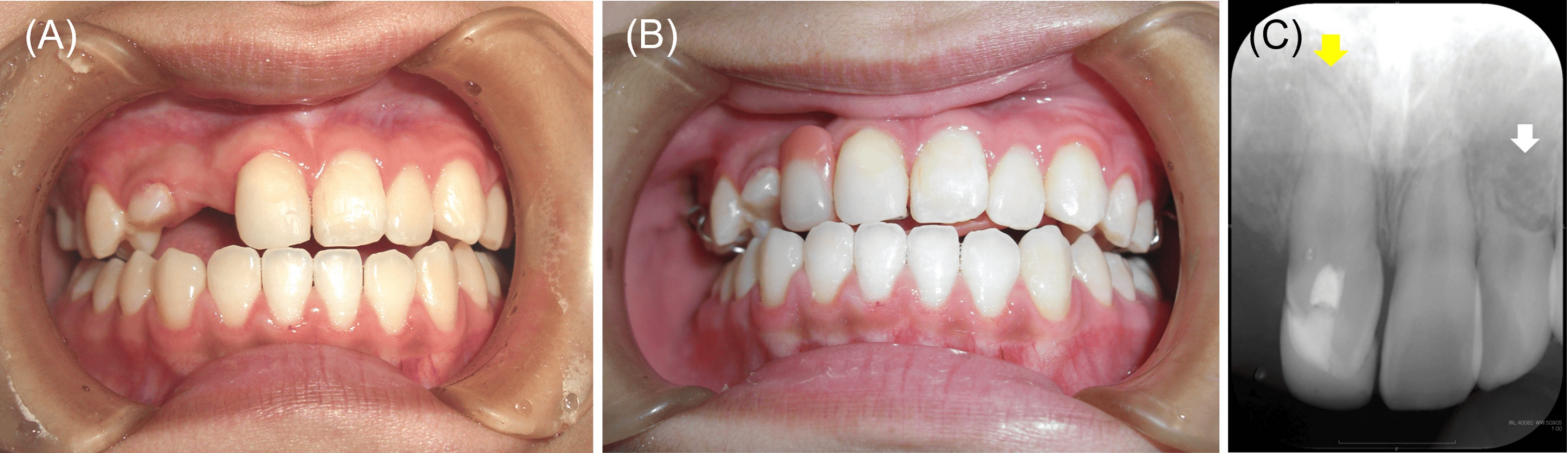
Two years and five months after the initial examination. (A) Intraoral photograph before wearing the denture. (B) Intraoral photograph wearing the denture. (C) Periapical radiograph of the maxillary right canine after crown restoration. The yellow arrow indicates maxillary right canine. The white arrow indicates maxillary left lateral incisor, with more than half of root resorption.

Immediately after this, the maxillary right lateral incisor was extracted. The patient was given an active plate with an auxiliary spring to move the erupted canine mesially; this resulted in a 3 mm shift after two months. In December 2018, composite resin (Clearfil Majesty® ES-2; Kuraray Noritake Dental, Niigata, Japan) was used to restore this canine after direct pulp capping (TheraCal PT®; Morimura, Tokyo, Japan) (Figure 4).

We considered restorative procedures that would avoid pulp exposure of this canine as much as possible; however, ultimately, pulp exposure was unavoidable. We performed occlusal adjustments of the denture as necessary to prevent occlusal interference between the maxillary right lateral incisor and the opposing tooth.

In April 2019, the maxillary right deciduous canine was extracted because the patient complained of spontaneous tooth pain due to severe mobility, and her denture was repaired to add the canine.

**Figure 5:**
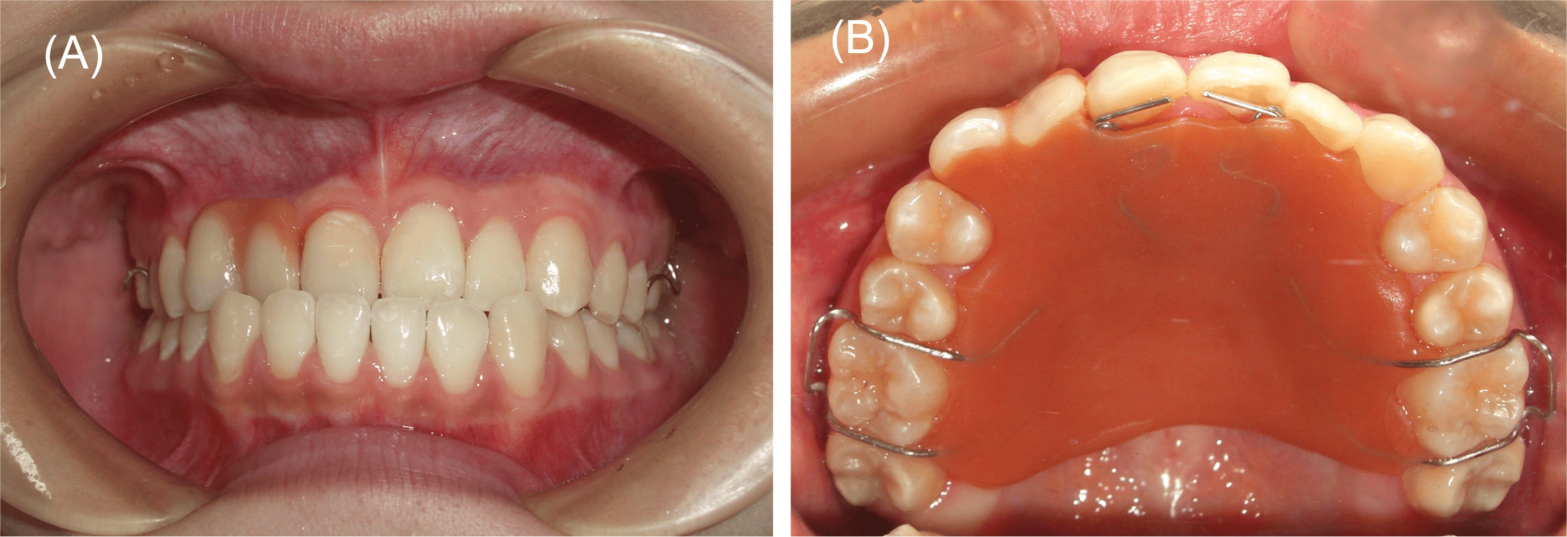
Intraoral photographs with removable denture placement six years after the initial examination. (A) Anterior occlusion. (B) Upper arch.

**Figure 6:**
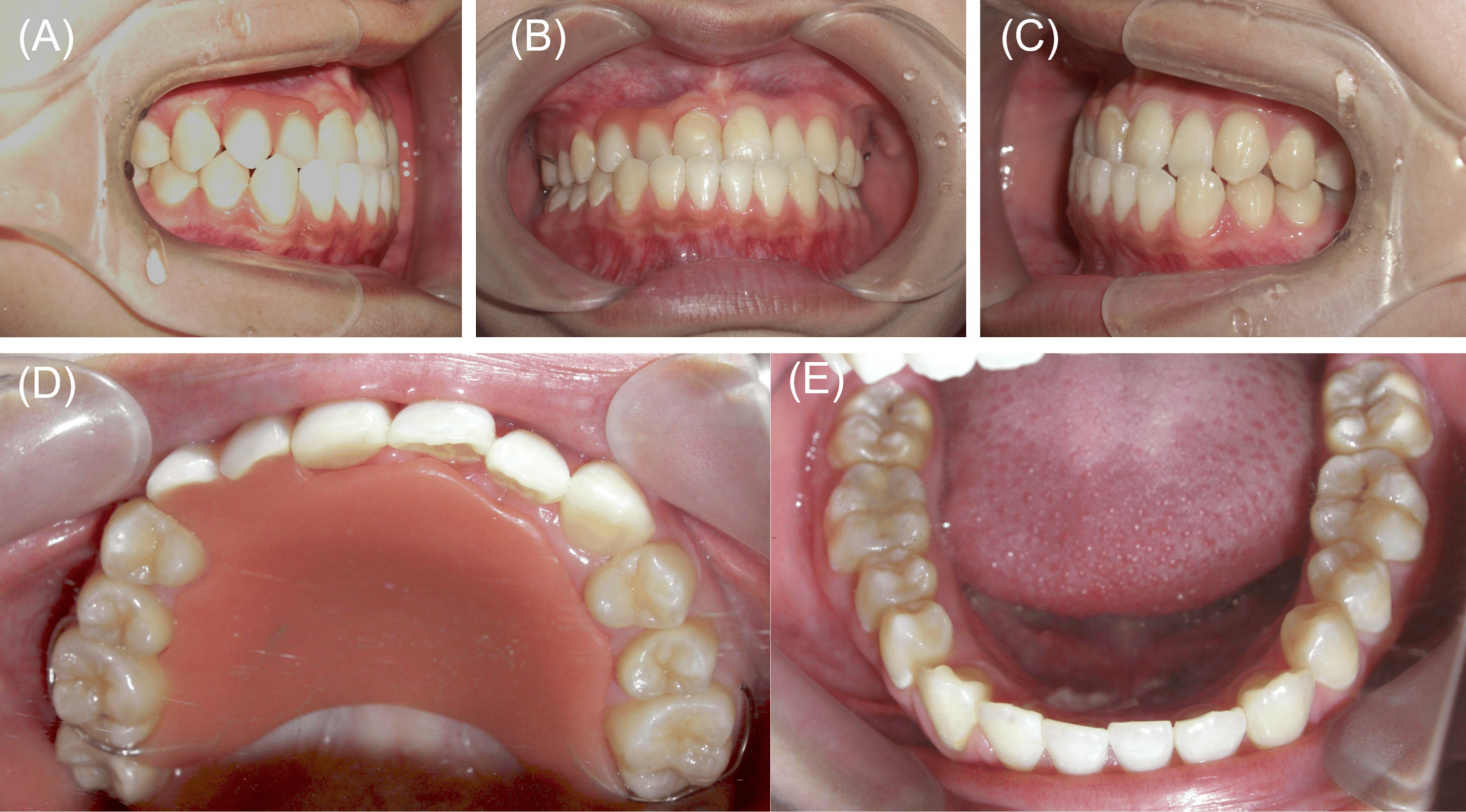
Intraoral photographs with removable denture placement eight years after the initial examination. (A) Right occlusion. (B) Anterior occlusion. (C) Left occlusion. (D) Upper arch. (E) Lower arch.

In August 2024, the mobility of the maxillary left lateral incisor was within the physiological range. Between July 2022 and August 2024, the condition of her teeth and dentition and their occlusal relationships had not significantly changed from when she first wore the dentures (Figures 5, 6).

**Figure 7:**
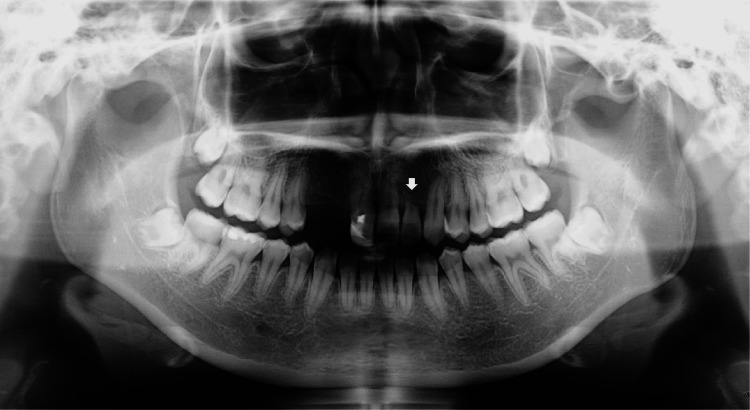
Panoramic radiograph 8 years after the initial examination (at age 19). The white arrow indicates the left maxillary lateral incisor. No significant changes in the degree of left maxillary lateral incisor root resorption were shown compared to the findings 10 months after the first consultation (Figure 3).

The panoramic radiograph revealed no significant changes in the degree of left maxillary lateral incisor root resorption compared to the findings in May 2017, 10 months after the first consultation (Figures 3, 7).

In the interview at that time, she responded that her anxiety had disappeared through the treatment. Furthermore, she told us that her sociable personality had improved. In the future, a fixed prosthesis will be implanted if she wishes.

## Discussion

Many studies have shown that the early diagnosis of an impacted canine and evaluation of the risk factors for incisor root resorption is crucial to prevent future complications, including severe root resorption of permanent incisors [5,8,12,14]. Chaushu et al. reported that females with dental follicles of the maxillary canines wider than 2 mm and normal lateral incisors are at greater risk for severe incisor root resorption associated with impacted or ectopic eruption of maxillary canines. In that study, univariate analyses also showed that severely mesiodistally displaced vertically positioned canines in the middle third of the adjacent incisor root were also factors, although this result was nonsignificant in multivariate analyses [5]. In our case, female patients and normal forms of lateral incisors were consistent with their study. We did not have the opportunity to evaluate the dental follicle width, position, and inclination angle of the impacted maxillary canines. To predict root resorption of maxillary incisors, it is essential to perform regular radiographic examinations, as such resorption does not always cause symptoms [12]. Indeed, the resorption in our patient did not cause symptoms. Therefore, unless dentists examine all pediatric patients regularly over a long period, the detection of such abnormalities will inevitably be delayed.

Another reason to suggest long-term follow-up of pediatric patients is to support the patient’s mental growth. A British study reported that irregular dental visits to treat undesired symptoms are strongly associated with dental anxiety in 9-year-old children and suggested that strategies be developed to promote regular, proactive dental visits [15]. Another study suggested that the psychological and social impacts of dental trauma can impair social functioning, emotional balance, and the well-being of children and suggested that long-term follow-up and monitoring are essential, particularly in growing patients whose dentition is still developing [16]. In our case, when the patient first came to our hospital, she was extremely shocked when faced with the sudden, unexplained loss of her permanent incisors. However, during the course of her treatment, she progressed from elementary school to high school, and both her oral and mental conditions significantly improved. We demonstrated that pediatric dentists who are often faced with such cases need to pay attention not only to the technical aspects of dental care but also to psychological consequences.

## Conclusions

In this report, we described a case of the eight-year management of ectopic eruption of the maxillary right canine with loss of the maxillary right central and lateral incisors due to severe root resorption in an 11-year-old girl. The patient wanted minimally invasive treatment. Therefore, we developed a strategy with the patient and her parents and treated the anomaly. When the patient first came to our hospital, she was extremely shocked. However, we relieved her mental distress over an eight-year period. Pediatric dentists must perform regular, long-term examinations of pediatric patients to detect dental abnormalities early and intervene if needed. Simultaneously, they should develop close relationships with patients and consider the psychological impact of damaged or lost teeth.
